# Assessment of Metallurgical and Mechanical Properties of Welded Joints via Numerical Simulation and Experiments

**DOI:** 10.3390/ma15103694

**Published:** 2022-05-21

**Authors:** Paolo Ferro

**Affiliations:** Department of Engineering and Management, University of Padova, Stradella San Nicola 3, 36100 Vicenza, Italy; paolo.ferro@unipd.it

Welding has been the most important joining technique applied to metallic materials since the early twentieth century when arc welding was introduced. Since then, new technologies have been developed, from high-energy density to solid-state welding processes, up to, most recently, hybrid metal extrusion and bonding. The advantages of welding are well known—the absence of weakening holes, the reduction of production cost, faster speed of fabrication if compared to bulky riveted/butted joints, etc. However, there are disadvantages, as well. Near the joint, the metallic material is altered, most of the time in a negative direction. Fatigue strength is reduced due to metallurgical and geometrical effects. Residual stresses are induced that, according to their ‘sign’, could further reduce the load-bearing capacity and the fatigue strength of the joint, above all in the high cycle fatigue regime. In this scenario, welding process numerical simulation is extremely useful to optimize the process parameters affecting the microstructure and the mechanical properties of the welded joint, avoiding expensive and time-consuming experiential trials. On the other hand, a static and fatigue strength assessment of welded joints through experiments is required to validate both numerical models and new design methodologies, such as the strain energy density [1] or the peak stress approach [2].

This Special Issue collects original works dealing with new advances in welded joints microstructure and mechanical characterization via numerical simulation and/or experiments.

A set of works are oriented on analytical modelling. Hybrid metal extrusion and bonding (HYB), a novel solid-state welding method for metals and alloys that utilizes continuous extrusion as a technique to enable filler metal additions, is modelled using a semi-analytical approach [3], allowing process parameter optimization in real-time. The same methodology is then used to rapidly calculate residual stresses in dissimilar S355–AA6082 butt welds produced by the same welding technique (HYB) [4]. Despite the advantages of analytical methods, numerical simulation is irreplaceable when the accurate prediction of thermal and residual stress is required. This is the case in which asymptotic residual stress distributions, which originate at the weld toe, needed to be captured and implemented in fatigue strength design approaches [5]. Or the case in which [6] the intriguing interactions between multi-pass welding and solid-state phase transformation need to be studied in view of a better residual stress control. Finally, numerical simulation can be used to better calibrate the experimental measurements, and vice versa [7], or to develop more accurate material models to be implemented in numerical simulations [8].

The new challenges in the welding sector are also aimed at the weldability of materials that could have been thought of as not weldable [9], or at the welded joint properties improvement [10] by a proper selection of the welding process and/or post-welding heat treatment.

Dissimilar welding is another topic covered. Friction stir processing on CuZn37/AA5056 is performed to study structural and phase evolution, with particular attention paid to intermetallic compound formation [11]. Finally, the laser/PEEK interaction is faced both experimentally and numerically to study the effect of pulse width and peak power on the geometrical morphology of the resulting hole [12].

Where are the studies on materials and welding processes oriented today? The design of new alloys for recycling and weldability with a low criticality index [13] is certainly a priority that requires new research works and advanced methodologies. In this context, the introduction of artificial intelligence used not only to design new alloys for sustainability, but also for the optimization of welding process parameters, is clearly a new way to go.

I believe that this collection of papers of high scientific value could help all people working in the welding sector who need to keep up to date with the recent developments. Artificial intelligence will be the next frontier, but it will be useless without a solid knowledge of the welding process based on modelling and experimental analysis.
